# How Do Phenolic Acids Change the Secondary and Tertiary Structure of Gliadin? Studies with an Application of Spectroscopic Techniques

**DOI:** 10.3390/ijms23116053

**Published:** 2022-05-27

**Authors:** Renata Welc, Rafał Luchowski, Konrad Kłosok, Wiesław I. Gruszecki, Agnieszka Nawrocka

**Affiliations:** 1 Institute of Agrophysics, Polish Academy of Sciences, Doświadczalna 4, 20-290 Lublin, Poland; k.klosok@ipan.lublin.pl (K.K.); a.nawrocka@ipan.lublin.pl (A.N.); 2 Department of Biophysics, Institute of Physics, Maria Curie Skłodowska University, 20-031 Lublin, Poland; rafal.luchowski@mail.umcs.pl (R.L.); wieslaw.gruszecki@mail.umcs.pl (W.I.G.)

**Keywords:** gliadin, phenolic acids, secondary structure, time-resolved fluorescence, FTIR technique

## Abstract

The effect of the chemical structure of selected phenolic acids on the molecular organization of gliadins was investigated with the application of Fourier Transform Infrared (FTIR) technique, steady-state, and time-resolved fluorescence spectroscopy. Hydroxybenzoic (4-hydroxybenzoic, protocatechuic, vanillic, and syringic) and hydroxycinnamic (coumaric, caffeic, ferulic, sinapic) acids have been used as gliadins modifiers. The results indicated that hydroxybenzoic acids due to their smaller size incorporate into spaces between two polypeptide chains and form a hydrogen bond with them leading to aggregation. Additionally, syringic acids could incorporate into hydrophobic pockets of protein. Whereas hydroxycinnamic acids, due to their higher stiffness and larger size, separated polypeptide chains leading to gliadin disaggregation. These acids did not incorporate into hydrophobic pockets.

## 1. Introduction

Gliadins, the major component of wheat gluten, are a heterogenous mixture of monomeric, globular proteins with similar amino acid sequences. They are classified as α-/β-, γ-, and ω-gliadins according to their electrophoretic mobility at acid pH [1]. The α-/β- and γ- gliadins have a similar molecular weight (30–35 kDa) and contain mainly α-helices and β-sheets, whereas ω-gliadins are dominated by β-turns and their molecular weight is 44–88 kDa. All fractions have extremely low solubility in an aqueous solution caused by the presence of disulphide bonds and hydrophobic interactions, which lead the protein chains to adopt a folded shape [2].

Polyphenols are compounds that naturally occur in plants, and are responsible for their color and characteristic taste. Polyphenols, as strong antioxidants, efficiently scavenge free radicals, thus helping to prevent neurological, cardiovascular, or intestinal diseases [3,4]. Due to a lot of beneficial biological functions, dietary polyphenols have attracted the attention of consumers looking for healthy food products [5].

Interactions between polyphenolic compounds and gluten proteins were intensively investigated in bread as well as in the model dough during mixing. Rheological and spectroscopic studies indicated that polyphenol molecules are prone to incorporate into the gluten network. The incorporation leads to changes in the secondary structure of gluten proteins and thus influences the quality of wheat dough and wheat bread [6,7,8,9]. Interactions between gliadins extracted from wheat dough and polyphenols were also investigated. The results showed that addition of anthocyanins caused abnormal folding or aggregation of gliadin proteins [10,11,12]. To ensure high protein affinity, polyphenol molecules should be small enough to penetrate inter-fibrillar regions of a protein. Simultaneously, they should be large enough to crosslink peptide chains at more than one point [13].

The complexity of the gluten system made the mechanism of interactions between gluten proteins and polyphenols unknown. It is difficult to determine which gluten protein fraction (gliadins or glutenins) interact with a phenolic acid. Additionally, gliadins are regarded as one of the strongest plant allergens. Hence, biochemical modification of gliadin can be one of the most important factors leading to reduction of its allergenicity. The most promising are studies in which antioxidants were used to modify gluten proteins [11,12]. The objective of this work was to investigate the proteins—polyphenols interaction using a simple model system containing only commercially available wheat gliadins, and selected phenolic acids belonging to the hydroxybenzoic and hydroxycinnamic groups.

From the structural point of view, hydroxycinnamic acids differ from hydroxybenzoic only in the presence of one additional double bond between the carboxyl group and the aromatic ring. We have attempted to connect changes in the structure of gliadins with the presence of individual functional groups on the aromatic ring of the acids. Phenolic acids belonging to the hydroxybenzoic and hydroxycinnamic groups have been arranged in pairs, and have the same substituents at the aromatic ring, but they differ in the length of the hydrocarbon chain (see Figure S1 in the Supplementary Material). Gliadins—phenolic acids complexes, were investigated with an application of Fourier transform infrared technique and steady-state as well as time-resolved fluorescence spectroscopy. The following acids were analyzed: hydroxycinnamic acid derivatives (caffeic, ferulic, coumaric, and sinapic acid) and hydroxybenzoic acid derivatives (protocatechuic, vanillic, 4-hydroxybenzoic, and syringic acid).

## 2. Results

### 2.1. FTIR

FTIR is a powerful analytical technique to investigate protein secondary structure and local conformational changes. A typical protein infrared spectrum contains nine characteristic vibrational bands (amides A, B, and I–VII) assigned to protein backbone and amino acid side chains vibrations. Amide I band (1720–1570 cm^−1^), which is the most sensitive vibrational band, was used to determine secondary structure of proteins.

**Figure 1:**
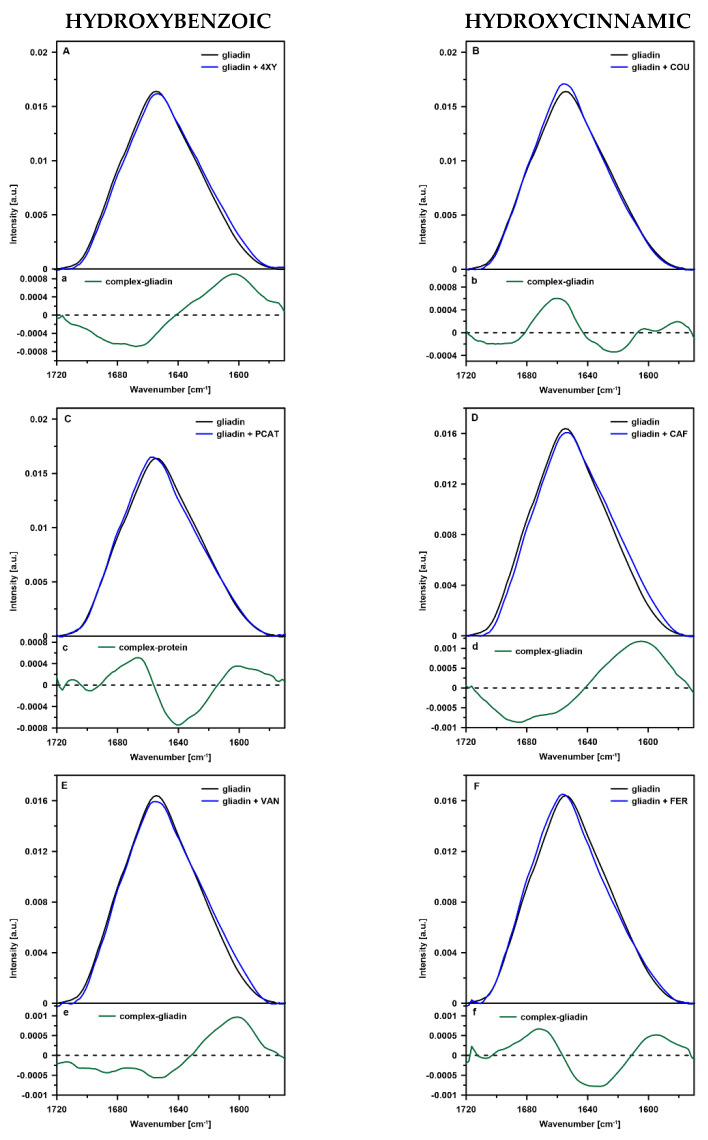

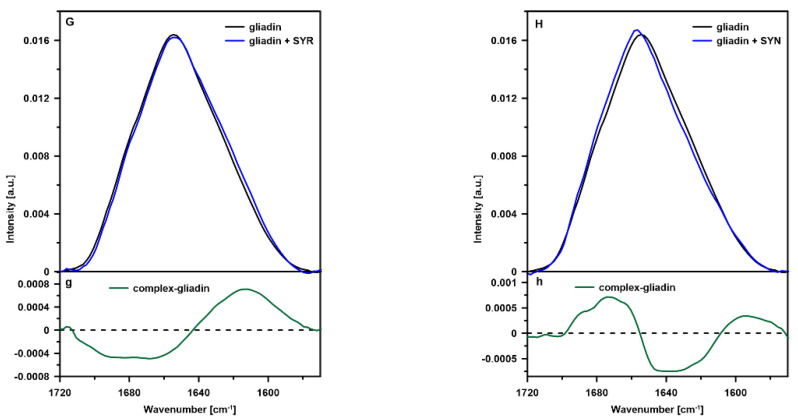
FTIR spectra of the gliadin and complexes of gliadin with phenolic acids: 4-xydroxybenzoic (4XY), coumaric (COU), protocatechuic (PCAT), caffeic (CAF), vanillic (VAN), ferulic (FER), syringic (SYR), and sinapic (SYN). Panels (**A**–**H**) present the original spectra and panels (**a**–**h**) present the difference spectra. The original, surface-normalized spectra are presented in the amide I region (1570–1720 cm^−1^).

Amide I band was analyzed to determine changes in the secondary structure of gliadin structure induced by phenolic acids belonging to two groups: hydroxycinnamic and hydroxybenzoic acids. Amide I bands of gliadin (control sample) and gliadin modified by selected phenolic acids are shown in Figure 1. The difference spectra, obtained by subtraction of gliadin spectrum from spectra of gliadin modified by phenolic acids, are presented in the lower panels of Figure 1.

Analysis of the difference spectra indicate that all phenolic acids caused changes in the secondary structure of gliadin proteins, and the type of changes depends on the group to which the acids are classified—hydroxybenzoic or hydroxycinnamic.

Most of the difference spectra of gliadin samples containing phenolic acids belonging to a hydroxybenzoic group (4-XY, VAN, and SYR) show a positive strong band in the spectral region between 1570 and 1640 cm^−1^, and a negative band between 1640 and 1720 cm^−1^. The first region corresponds to antiparallel-β-sheets (ca. 1630 cm^−1^), aggregated β-structures such as pseudo-β-sheets (ca. 1620 cm^−1^), and hydrogen-bonded antiparallel-β-sheets (ca. 1620 cm^−1^). Whereas the positive bands are associated with α-helices (ca. 1650 cm^−1^), β-turns (ca. 1666 cm^−1^), and β-sheets (1680 cm^−1^) [14,15,16].

As can be seen, in the presence of 4-XY, VAN, and SYR acids, gliadins contain a smaller number of all basic secondary structures (α-helices, β-turns, and β-sheets) compared to the control sample. Simultaneously, the amount of aggregated structures and antiparallel-β-sheets increases significantly. According to the authors of [17], pseudo-β-sheets are stabilized by strong, intermolecular hydrogen bonds. It can be concluded that the presence of 4-XY, VAN, and SYR promotes the formation of these types of bonds in gliadin polypeptide chains. It is also reported that intensive band at 1611–1630 cm^−1^ can be connected with fibrillation of native globular proteins rich in β-sheets and amyloid formation [18]. Parallel or antiparallel β-sheets can assemble into a cross β-structure in which an inter-strand and inter-sheet distance is about 5 Å and 6–12 Å, respectively [19]. Due to smaller molecular size compared to hydroxycinnamic acids, hydroxybenzoic acids could be incorporated into the amyloid structure and located in the inter-strand spaces. Such a location of the acids could allow them to form hydrogen bonds with adjacent strands within cross β-structure, which, in turn could lead to the protein structure tightening and aggregation. Interestingly, PCAT acid-induced different changes in the secondary structure of gliadins compared to the other acids belonging to the same group. In this case, the number of β-turns and β-sheets increased at the expense of antiparallel-β-sheets and hydrogen-bonded β-turns (1643 cm^−1^).

Interactions between gliadins and hydroxycinnamic acids (except for caffeic acid) resulted in opposite changes in the secondary structure of protein compared to the group of hydroxybenzoic acids. In these systems, a smaller number of unordered aggregated structures (1600 cm^−1^), and a significant reduction of hydrogen-bonded β-turns (1640 cm^−1^) and hydrogen-bonded antiparallel β-sheets (1627 cm^−1^) were observed. Simultaneously, an increase in the amount of basic secondary structures: α-helices (1650 cm^−1^), β-turns (1666–1677 cm^−1^), and β-sheets (1666–1677 cm^−1^) were detected. This result indicates that interactions between gliadin and hydroxycinnamic acids lead to the loosening of the polypeptide chains and disaggregation of the protein. The same type of changes in the secondary structure of gliadins was observed in the case of the gliadin-PCAT acid system, whereas caffeic acid-induced changes were characteristic for hydroxybenzoic acids. Disaggregation of gliadins can be explained based on the possibility of gliadin amyloid formation and incorporation of phenolic acids between polypeptide chains. In contrast to the hydroxybenzoic acids, hydroxycinnamic acids contain an additional fragment of the hydrocarbon chain with a double C=C bond in their structure. This double bond is conjugated with an aromatic ring which leads to an increase in the stiffness of the acid molecule. Rigid and larger than hydroxybenzoic acids molecules of hydroxycinnamic acids could be able to break hydrogen bonds between polypeptide chains and thus separate them. It is reported that a lot of polyphenolic compounds (including ferulic acid) can modulate or even inhibit the process of amyloid beta Aβ aggregation [20]. The mechanisms by which polyphenols affect aggregations are connected with covalent and non-covalent bond formation, and charge transfer between polyphenol and the backbone or side-chain amino acids [21,22]. It seems probable that phenolic acids belonging to the hydroxycinnamic group are located in the inter-sheet space of gliadin cross β-structure. This location would allow acid molecules to form hydrogen bonds with amino acids located on opposite sides of the cross beta spines.

### 2.2. Time-Resolved Fluorescence

Proteins contain three aromatic amino acids which are responsible for their natural ultraviolet fluorescence: tyrosine, phenylalanine, and tryptophan. Due to efficient excitation energy transfer, the emission of protein is dominated by tryptophan. Tryptophan residues are highly sensitive to the local environment and show significant spectral shift, for example, as a result of the formation of the hydrogen bond. Intrinsic fluorescence of proteins as well as fluorescence decay kinetics can provide important information about protein structure and dynamics. These techniques are often used to study protein folding as well as association reactions [23].

**Figure 2:**
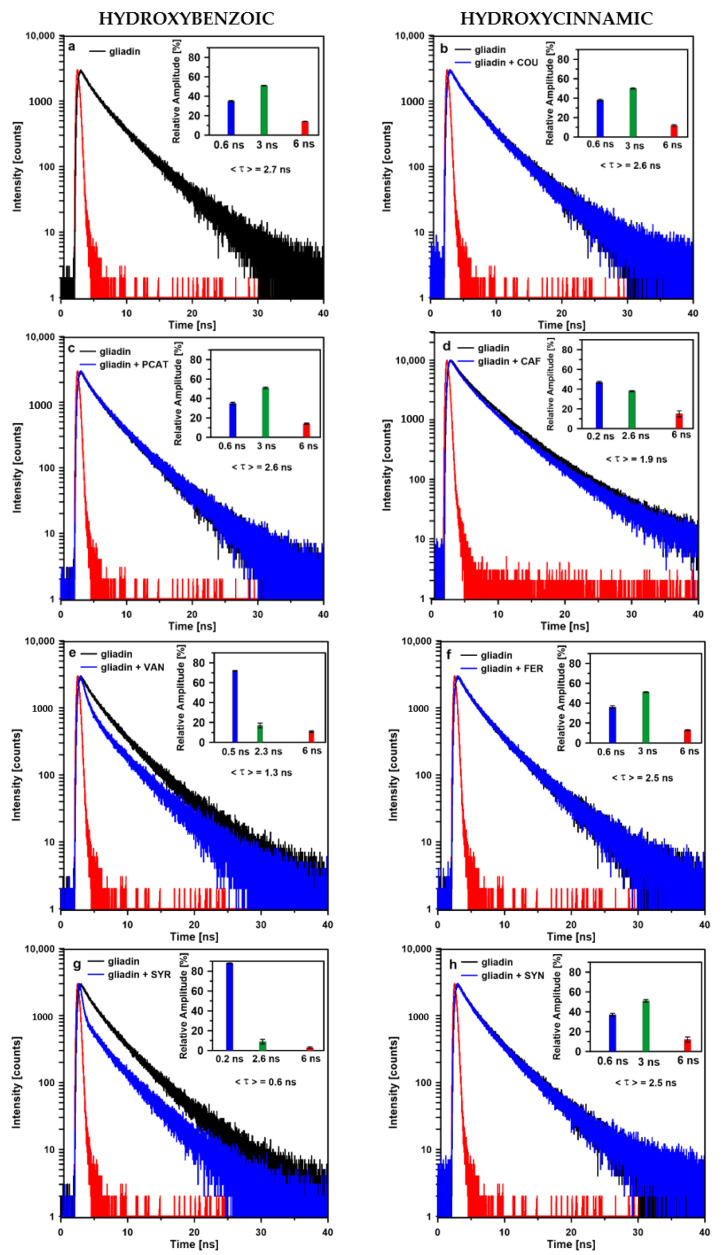
Fluorescence decay kinetics of tryptophan emission in the gliadin (**a**) and gliadin-phenolic acids complexes (**b**–**h**). Intensity weighted average lifetime <τ> values are presented in the figure. The fluorescence decay kinetics were fitted with three components characterized by appropriate lifetimes for individual samples. The inner panels present relative amplitudes of the fluorescence lifetime components.

Fluorescence decay kinetics of tryptophan emission and intensity weighted average fluorescence lifetime <τ> values recorded for the gliadin without phenolic acid as well as gliadin-phenolic acids complexes are presented in Figure 2. The fluorescence decay kinetics were fitted with three components characterized by appropriate lifetimes for individual samples. The inner panels in Figure 2 present relative amplitudes of the fluorescence lifetime components.

As can be seen, the average fluorescence lifetime of tryptophan in protein without addition of acids was 2.7 ns. In this system, three lifetime components: τ_1_ = 6 ns, τ_2_ = 3 ns, and τ_3_ = 0.6 ns were found. It is reported that two lifetime components (0.4–0.5 ns and 2–4 ns) are characteristic for free tryptophan in water solution [24]. Since ethanol-water solution (70:30, *v*/*v*) was used as a solvent of gliadin and phenolic acids, fluorescence decay kinetic from free tryptophan in this solution was recorded (see Figure S2 in the Supplementary Material). Two lifetime components were observed: τ_1_ = 0.4 ns (6%) and τ_2_ = 2.3 ns (94%). In general, fluorophores in proteins were characterized by a longer fluorescence lifetime in comparison with free fluorophores dissolved in solvents [23]. Therefore, lifetime components: 0.6 ns and 3 ns observed for tryptophan in a gliadin environment can be assigned to tryptophan molecules that are exposed to an ethanol-water environment (hydrophilic). Figure 2a presents the distribution of the amplitudes of the tryptophan lifetime components in gliadins. As can be seen, the highest values of amplitudes correspond to 3 ns and 0.6 ns component (51% and 35%, respectively), which indicates that most of the tryptophan molecules are exposed to the solution (hydrophilic environment).

This result is in agreement with Stevenson and Preston [25] who, based on intrinsic fluorescence and quenching studies of gliadin extracts, showed that a significant part of tryptophan residue is located at the surface of a protein or in polar regions. The presence of the longest lifetime component of tryptophan in gliadins (6 ns) is probably connected with tryptophan molecules located inside the protein complex, which do not interact with the solvent environment. The relative amplitude of this component is 14%.

The addition of syringic and vanillic acids (hydroxybenzoic group) resulted in a shortening of the average fluorescence lifetime of gliadins from 2.7 ns to 0.6 ns and 1.4 ns, respectively. In the case of vanillic acid, fluorescence lifetime components were shortened from 3 ns to 2.3 ns and from 0.6 ns to 0.5 ns. Additionally, the amplitude of the shortest component increased from 35% to 72%. The amplitude of the longest component decreased by about 5%.

In the case of a complex gliadin-syringic acid lifetime, component 0.5 ns is shortened to 0.2 ns and its amplitude increased to 89%. Such an observation showed that the gliadin–VAN and gliadin–SYR complexes’ formation ensures efficient quenching of tryptophan fluorescence. Moreover, the shortest value of tryptophan average fluorescence lifetime observed in the presence of SYR acid indicated that syringic acid interacts stronger with gliadins compared to vanillic acid. The shortening fluorescence lifetime of gliadin could be the result of the attachment of VAN and SYR acid molecules to amino acids in close proximity to tryptophan. Overlapping the electron levels of tryptophan and phenolic acids allows for efficient excitation energy transfer between these molecules. Additionally, a reduction in the lifetime amplitude assigned to tryptophan residues exposed to the ethanol environment indicated that these phenolic acids were located mainly on the gliadins’ surface. On the other hand, tryptophan excitation energy quenching can be a result of protein aggregation leading to converting excitation energy into heat [23]. In the case of gliadin–SYR and gliadin–VAN systems, this mechanism is more probable because the process of gliadin aggregation in the presence of hydroxybenzoic acids was confirmed by the FTIR technique. Moreover, a decrease in the amplitude of the longest component (6 ns), especially in the case of SYR acid, was observed. This result may indicate that SYR acid can incorporate into hydrophobic pockets and quench tryptophan excitation energy.

PCAT acid, belonging to the same group as 4XY, VAN, and SYR, does not significantly affect the value of the average fluorescence lifetime of tryptophan in gliadins. However, the analysis of the amide I band showed that this acid induced different changes in the protein secondary structure compared to the other acids within this group. Additionally, it was not possible to register tryptophan fluorescence decay kinetic in the gliadin–4XY system due to the fact that the acid fluorescence spectra overlapped with the spectra of gliadins at the wavelength where emission was observed.

Phenolic acids belonging to a hydroxycinnamic acid group (except for caffeic acid) did not affect the average fluorescence lifetime of tryptophan in gliadins compared to the control sample. The relative amplitudes of individual lifetime components were similar to those calculated for the tryptophan in the control sample. However, the analysis of the amide I band showed gliadin disaggregation in the presence of these acids. No significant reduction in the average fluorescence lifetime of tryptophan observed in the presence of hydroxycinnamic acid suggests that COU, FER, and SYN acids are located within the inner part of gliadin. Such a location would prevent excitation energy transfer from surface tryptophan towards phenolic acid molecules.

In the case of caffeic acid, the average fluorescence lifetime of protein decreased from 2.7 ns to 1.9 ns and the amplitude of the shortest component increased from 35–47%. Additionally, the FTIR technique confirmed gliadins aggregation in the presence of this acid. As can be noticed, caffeic acid-induced changes in the gliadin structure were characteristic for hydroxybenzoic acids.

### 2.3. Steady-State Fluorescence

**Figure 3:**
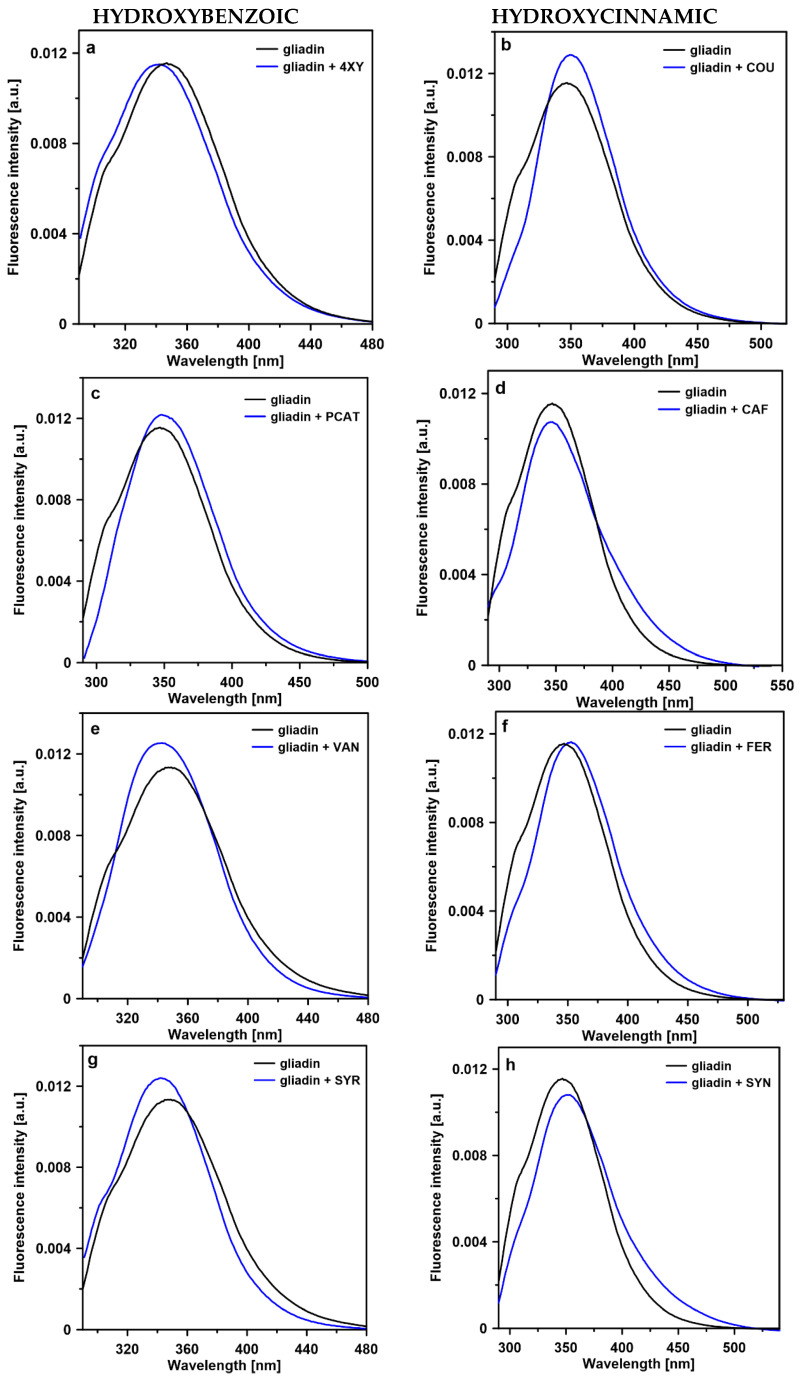
Fluorescence emission spectra recorded from gliadin and complexes of gliadin with phenolic acids: 4-xydroxybenzoic (4XY), coumaric (COU), protocatechuic (PCAT), caffeic (CAF), vanillic (VAN), ferulic (FER), syringic (SYR), and sinapic (SYN) (**a**–**h**). The excitation wavelength was set at 280 nm. The spectra were normalized to get the same area beneath each spectrum.

Phenolic acid-induced changes in the tertiary structure of gliadins were also analyzed using steady-state fluorescence spectroscopy. Figure 3 presents steady-state fluorescence emission spectra recorded for gliadin and protein-phenolic acid mixtures. The emission spectrum of gliadin displays a wide band centered at 348 nm. As can be seen for gliadins–hydroxybenzoic acids (except for PCAT) systems, the maximum of fluorescence spectra was shifted towards shorter wavelengths (blue shift) compared to the gliadin emission band. The strongest effect (6 nm) was observed for syringic acid. The blue shift of the tryptophan fluorescence emission spectrum indicated that the microenvironment of tryptophan residues became more hydrophobic [23]. Interactions between proteins and phenolic compounds were also investigated by several research groups. The authors of [26] observed a blue shift of protein emission spectrum as a result of hydrogen bonds formation between gliadin and quercetin. A shift towards a shorter wavelength was also observed when BSA was titrated with two flavones (quercetin and kaempferol) [27], and when milk β-lactoglobulin formed complexes with tea polyphenols [28]. A blue shift of the protein spectra observed in the presence of hydroxybenzoic acids can be connected with hydrogen bond formation or/and hydrophobic interactions between these acids and gliadins. These intermolecular interactions may lead to the protein tightening or/and aggregation and disordered structure formation. The process of protein aggregation in the presence of hydroxybenzoic acid derivatives was proved by analysis of the amide I band. Additionally, the time-resolved spectroscopy technique confirmed that SYR and VAN acids were responsible for effective tryptophan excitation energy quenching as a result of aggregated structure formation. The highest blue shift value observed for syringic acid corresponded to the most efficient fluorescence quenching. In the case of gliadin mixed with PCAT acid, a slight shift (4 nm) of emission spectra maximum towards longer wavelength compared to the protein emission spectra was detected. Phenolic acids belonging to a hydroxycinnamic group (except for CAF) interacted with gliadins differently. As can be seen, the maximum fluorescence emission spectra of protein was shifted towards longer wavelengths in the systems with COU, FER, and SYN acids. The red shift of gliadin emission indicated that in the presence of these acids, the tryptophan microenvironment became more hydrophilic, and tryptophan residues were more exposed on the protein surface [23]. The same effect was observed in the case of PCAT acid. The change in the gliadin polarity may be the result of protein disaggregation or/and unfolding, leading to the exposition of tryptophan residues which were located in deeper parts of the protein. This result is in agreement with data obtained from time-resolved fluorescence and FTIR spectroscopy.

## 3. Materials and Methods

### 3.1. Materials

Gliadins, protocatechuic, p-coumaric, caffeic, 4-hydroxybenzoic, vanillic, and ferulic acids were purchased from Sigma Aldrich (Poznań, Poland). Syringic and sinapic acids were purchased from Alfa Aesar (Gdańsk, Poland) and Apollo Scientic (Manchester, UK), respectively. All reagents were, at least, of analytical grade.

### 3.2. Samples Preparation

In order to form gliadin-phenolic acids complexes, protein, as well as phenolic acids, were dissolved in a 70% ethanol solution. Gliadins molar concentration was estimated based on their averaged molecular weight (40 kDa), and it was 5 μM. Due to the low molecular weight of phenolic acids in relation to gliadins, acids were added to the protein solution in a molar ratio of 10:1. In the next step, the mixture was incubated for 1 h at room temperature. After that, the possible aggregates were removed from the samples by centrifugation for 30 min at 14,000× *g*. Supernatant was collected and analyzed with an application of spectroscopic methods. Since the addition of acids caused a slight decrease in the pH of the solutions (by 0.2–0.3), the gliadin solutions (control sample) were prepared in the solution with a pH corresponding to the pH after phenolic acid addition.

All experiments were repeated at least three times. In the case of FTIR, the spectra presented in Figure 1 were averaged over three registered spectra.

### 3.3. Fourier Transform Infrared Spectroscopy

The FT-IR spectra were collected with a Nicolet 6700 FT-IR spectrophotometer (Thermo Scientific, Waltham, MA, USA) equipped with a single reflection diamond ATR cell. Samples were deposited at the diamond surface by evaporation under a nitrogen stream. Infrared absorption spectra were recorded in the region between 4000 and 400 cm^−1^ with a resolution of 4 cm^−1^. FT-IR spectra were analyzed with an application of ORIGIN software (v.9.0 PRO, OriginLab Corporation, Northampton, MA, USA). To determine changes in the secondary structure of gliadins, difference spectra in the amide I region were calculated. The spectrum of the control sample (gliadins) was subtracted from spectra of samples containing gliadins and phenolic acids.

### 3.4. Steady-State Fluorescence

Fluorescence emission spectra were collected using a FluoroMax-4P spectrofluorometer (Horiba Jobin Yvon, Kyoto, Japan). The excitation wavelength was set at 280 nm (tryptophan excitation) and the slits of both monochromators were set to 5 nm. Fluorescence emission spectra were analyzed with the application of ORIGIN software (v.9.0 PRO, OriginLab Corporation, Northampton, MA, USA). Steady-state fluorescence spectra were baseline corrected and normalized to have the same integrated area under each spectrum.

### 3.5. Time-Resolved Fluorescence

The fluorescence lifetime of samples was measured using FluoTime 300 spectrometer (PiqoQuant, Berlin, Germany). Excitation was set at 280 nm (characteristic for tryptophan) from the diode (PLS280) with a 10 MHz frequency of pulses. The time resolution was kept at 4 ps. Detection (at 310 nm) was performed with a microchannel plate and time-correlated single-photon counting (TCSPC) system PicoHarp 300. Observations were made through 300 long-wavelength pass filter (Semrock FF01-300/LP-25). The scattered light was removed by using a 300 short-wavelength pass filter (Semrock FF01-300/SP-25).

Fluorescence lifetime decays were fitted using FluoFit Pro software (PicoQuant, Berlin, Germany), deconvoluted with the instrument response function (IRF), and analyzed as a sum of experimental terms according to the equation:

$$I\left(t\right)=\underset{i}{\sum }\alpha_{i}\exp\left(\frac{-t}{\tau_{i}}\right)$$

where *τ_i_* are the decay time components and *α_i_* are the pre-exponential factors (amplitudes) of the individual components (∑*α_i_* = 1).

The quality of the fits was judged from the *χ*^2^ value (~1).

## 4. Conclusions

In general, the results obtained in the present experiment showed that the mechanism of interactions between phenolic acids and gliadins depends on the chemical structure of acids. Hydroxybenzoic acids, due to their smaller size and lower stiffness “fit” easily into spaces between two polypeptide chains, form hydrogen bonds with them, and tighten the entire structure of gliadins leading to aggregation. Steady-state and time-resolved fluorescence spectroscopy showed that the strongest impact on protein functionality had syringic acid. The presence of two methyl groups at the aromatic ring made SYR the most hydrophobic molecule within an analyzed hydroxybenzoic group. As a result, SYR may locate in deeper parts of the protein complex compared to the other acids belonging to the same group. Therefore, the amplitude of the lifetime component assigned to the tryptophan closed in hydrophobic pockets decreased in the presence of SYR acid. VAN acid containing only one methyl group reduced the amplitude of the longest lifetime component much less, compared to SYR acid. Probably most of the vanillic acid molecules were found between polypeptide chains.

The presence of an additional fragment of hydrocarbon chain containing a double C=C bond caused the hydroxycinnamic acids to induce different changes in the gliadin structure. The higher stiffness of these molecules, as well as their larger size allowed them to separate polypeptide chains. Amide I band analysis and steady-state fluorescence spectroscopy confirmed gliadin disaggregation. Despite higher hydrophobicity of SYN and FER, these acids did not incorporate into hydrophobic pockets.

Two of the studied phenolic acids—caffeic and protocatechuic—showed other mechanisms of interactions than phenolic acids included in the same group. Caffeic acid caused structural changes characteristic for hydroxybenzoic acids, whereas protocatechuic acid caused changes typical for hydroxycinnamic acids. The reasons for this type of behavior of both acids is difficult to explain at this stage of the study and require further detailed investigation.

## Data Availability

Not applicable.

## Supplementary Materials

The following supporting information can be downloaded at https://www.mdpi.com/article/10.3390/ijms23116053/s1. The fluorescence decay kinetics were fitted with components characterized by the lifetimes: 0.2 ns and 2.6 ns. The inner panel presents relative amplitudes of the fluorescence lifetime components.
