# Supplementary material for: How Do Phenolic Acids Change the Secondary and Tertiary Structure of Gliadin? Studies with an Application of Spectroscopic Techniques
Source: Int J Mol Sci. 2022 May 27;23(11):6053. doi: 10.3390/ijms23116053 (PMC9181179; doi:10.3390/ijms23116053)
Supplement: Supplementary file 1 [file ijms-23-06053-s001.zip › ijms-1727229-supplementary.pdf]

Supplementary Material

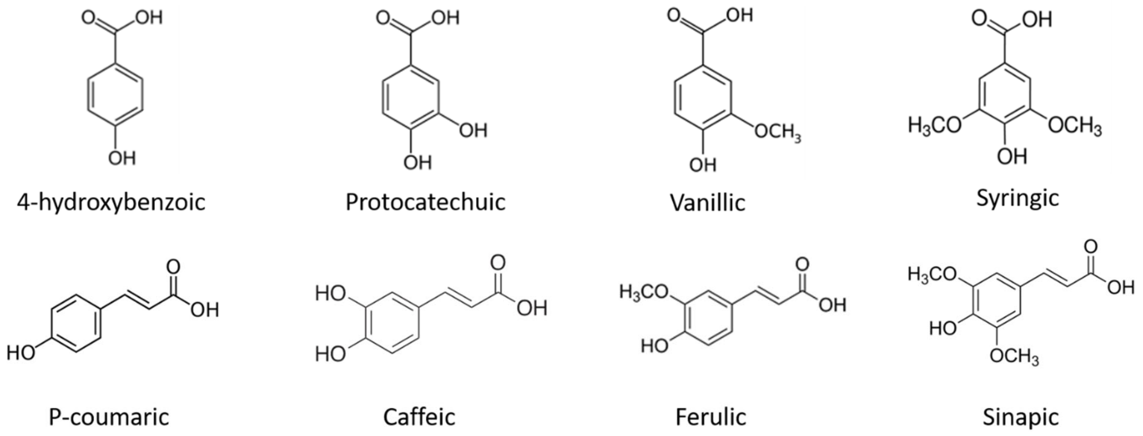

Figure S1. Chemical structure of analyzed phenolic acids.

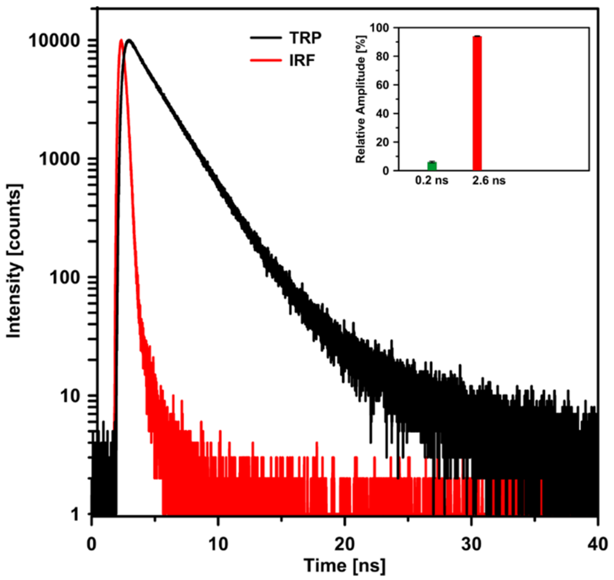

Figure S2. Fluorescence decay kinetics of tryptophan emission in ethanol-water (70:30 v/v) solution. The fluorescence decay kinetics were fitted with components characterized by the lifetimes: 0.2 ns and 2.6 ns. The inner panel presents relative amplitudes of the fluorescence lifetime components.
